# Where innovations flourish: an ethnographic and archaeological overview of hunter–gatherer learning contexts

**DOI:** 10.1017/ehs.2020.35

**Published:** 2020-06-17

**Authors:** Sheina Lew-Levy, Annemieke Milks, Noa Lavi, Sarah M. Pope, David E. Friesem

**Affiliations:** 1 Simon Fraser University, Department of Psychology, Burnaby, BC, Canada; 2 Department of Archaeology and Heritage Studies, Aarhus University, Aarhus, Denmark; 3 Institute of Archaeology, University College London, London, UK; 4 Department of Anthropology, University College London, London, UK; 5 Department of Comparative and Cultural Psychology, Max Planck Institute for Evolutionary Anthropology, Leipzig, Germany; 6 McDonald Institute for Archaeological Research, University of Cambridge, Cambridge, UK; 7 Zinman Institute of Archaeology, University of Haifa, Haifa, Israel

**Keywords:** Innovation, hunter–gatherers, childhood, adolescence, archaeology, child development

## Abstract

Research in developmental psychology suggests that children are poor tool innovators. However, such research often overlooks the ways in which children's social and physical environments may lead to cross-cultural variation in their opportunities and proclivity to innovate. In this paper, we examine contemporary hunter–gatherer child and adolescent contributions to tool innovation. We posit that the cultural and subsistence context of many hunter–gatherer societies fosters behavioural flexibility, including innovative capabilities. Using the ethnographic and developmental literature, we suggest that socialisation practices emphasised in hunter–gatherer societies, including learning through autonomous exploration, adult and peer teaching, play and innovation seeking may bolster children's ability to innovate. We also discuss whether similar socialisation practices can be interpreted from the archaeological record. We end by pointing to areas of future study for understanding the role of children and adolescents in the development of tool innovations across cultures in the past and present.

## Graphical abstract

**Figure:**
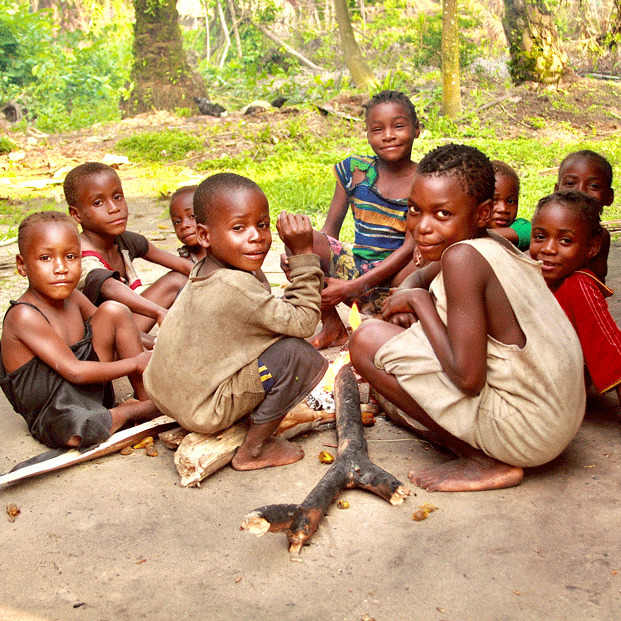

**Media summary:** Socialisation practices emphasised in hunter–gatherer societies may bolster child and adolescent innovativeness.

Both social learning and innovation are central to cumulative cultural evolution (Legare & Nielsen, 2015). However, while children in diverse societies demonstrate a suite of cognitive traits which make them especially sensitive to identifying and learning cultural information (Henrich & McElreath, 2003; Kline, 2015), psychologists working primarily in WEIRD societies (Western, Educated, Industrialised, Rich, and Democratic – Henrich et al., 2010) have noted that making novel tools is difficult for children under the age of 10 (Beck et al., 2011; Cutting et al., 2011; Nielsen et al., 2014; Whalley et al., 2017). Here, we argue that cross-cultural diversity in subsistence and socialisation practices complicates the image of children as infrequent tool innovators. Drawing upon the ethnographic literature, we argue that the developmental niche documented among several contemporary hunter–gatherer societies may set the stage for children's innovative capabilities to flourish. Elsewhere, we have argued that hunter–gatherer children have probably contributed to changes in cultural values (Reckin et al., in press). Here, we focus our discussion on children's potential and actual contributions to technological and/or material innovations in the past and present.

In what follows, we first describe the results of psychological studies on innovation, and outline how gaps in prior knowledge, including previous familiarity with the testing materials, and asocial experimental settings, may limit researchers’ ability to measure variation in children's tool innovation across cultural contexts. Second, we outline how the cultural and subsistence contexts of hunter–gatherers foster behavioural flexibility, including in childhood and adolescence. Third, we summarise findings from two meta-ethnographic reviews on learning in hunter–gatherer societies (Lew-Levy et al., 2017, 2018), focusing on specific socialisation practices which may encourage hunter–gatherer child and adolescent innovative capabilities. While many of these practices appear to be widespread, it is important to note that hunter–gatherers are diverse, and thus, not all descriptions will generalise to all hunter–gatherer societies. Wherever possible, we also outline clear ethnographic examples of children's innovations. Fourth, using examples from the Palaeolithic, Paleoindian, and Late Stone age, we illustrate how the ethnographic record can provide an interpretative framework for enriching how archaeologists think about, and identify, children as innovators. We also outline some of the challenges to identifying social learning processes in the archaeological record. Finally, we outline areas of future research for psychology, anthropology, and archaeology.

## Defining ‘innovation’ across disciplines

Innovation is central to the behavioural flexibility of human and non-human animals alike (Reader & Laland, 2001). Through innovations, species can rapidly respond to environmental novelty (Laland, 1992). In humans, innovations in social conventions, such as rituals, and instrumental skills, such as hunting technology, have helped our species inhabit diverse and challenging environments, and coordinate within and across social groups (Boyd & Richerson, 1985; Fogarty et al., 2015; Legare & Nielsen, 2015; McElreath et al., 2003). Because of its importance to human evolution and culture, the study of innovation spans several academic disciplines, and each discipline defines the term differently (Fogarty et al., 2015; Walsh et al., 2019). In the present paper, we draw upon definitions from psychology and archaeology.

In psychology, innovation, and specifically, tool innovation, has been defined as the construction of ‘new tools, or using old tools in new ways, to solve new problems’ (Legare & Nielsen, 2015, p. 689). Although innovative behaviours, including tool use and modification, have been described in other species (see Griffin & Guez, 2014 for review; Lefebvre et al., 2004; Overington et al., 2009; Reader et al., 2011), psychologists are often concerned with understanding the unique cognitive and developmental processes which underpin human innovation, including advanced analogical (Chan et al., 2011; Markman et al., 2011) or counterfactual reasoning (Tijus et al., 2009) to devise solutions, and inhibition (Gönül et al., 2018) or cognitive flexibility (Gönül et al., 2019; Pope et al., 2020) to apply them.

Archaeologists often separate innovation from invention, the latter defined as ‘a wholly new phenomenon’, and the former, as ‘a novel modification of something already in existence that proves adaptive and diffuses through a population by processes of selection’ (Walsh et al., 2019, p. 54; see also Richerson & Boyd, 2005; Mesoudi & O'Brien, 2008; Rogers, 1983; Shennan, 2001). Archaeologists are concerned with understanding the contexts under which a phenomenon has undergone observable adaptive change over time (Walsh et al., 2019). Usually, archaeologists are restricted to the study of innovations that have ‘spread in a population to a detectable frequency’ (Fogarty et al., 2015, p. 737; see also Walsh et al., 2019). Archaeological research typically emphasises the role of technological and cultural innovation in relation to the evolution of behavioural and cognitive complexity, the ability to thrive in new ecological niches, and/or the transmission and diffusion of cultural traits (e.g. Hussain & Will, in press; Knecht, 1991; McBrearty & Brooks, 2000; Shea & Sisk, 2010).

Building on these definitions, the present paper views innovations as creative outputs that are new and useful in specific settings (Fogarty et al., 2015), and which are transmitted throughout a group. We consider innovations as arising through multiple individual- and group-level processes, including invention, modification, recombination, trial-and-error and copying error (Ramsey et al., 2007). We discuss both the processes (i.e. socialisation practices) and products (e.g. new subsistence technologies) of child and adolescent innovators.

## Experimental research on children as innovators

Evidence of innovative capacities can be found early in human development. During this time, innovations serve an important role in supplementing knowledge or ability in both social and physical domains. For example, when learning to walk, infants develop unique strategies for using supports (Adolph & Robinson, 2013) and 2–3-year-olds fill gaps in their lexicon by combining or modifying known words, such as saying ‘I can button it’ rather than ‘I can turn it on’ (Clark, 1982). Throughout childhood, innovations are seen in children's pretence and game play (Carr et al., 2016; Nielsen et al., 2012) and during this time cognitive skills which probably support innovation improve drastically, including analogical reasoning (Richland et al., 2006, 2010), counterfactual reasoning (Rafetseder et al., 2013), inhibition (Montgomery & Koeltzow, 2010) and cognitive flexibility (Deák & Wiseheart, 2015; Doebel & Zelazo, 2015). Further, 5-year-old children outperform older children and adults in contexts which require them to consider causal relationships or object uses in innovative ways (Defeyter & German, 2003; Gopnik et al., 2015; Lucas et al., 2015).

Although creative problem-solving metrics like divergent thinking (Gönül et al., 2019), floating object (Cheke et al., 2012; Hanus et al., 2011; Nielsen, 2013) and functional fixedness (Defeyter & German, 2003) tasks have been conducted alongside or as proxies for innovation metrics (Carr et al., 2016; but see Beck et al., 2016), here we focus on direct measures of tool innovation. For children, the most prevalent tool innovation metric is the hook task, a paradigm originally devised for corvids (Bird & Emery, 2009; Weir et al., 2002) and later modified for humans (Beck et al., 2011; Cutting et al., 2011). In this paradigm, participants must bend a malleable stick, often a pipe cleaner, into a hook in order to retrieve a basket containing a sticker or other reward from the bottom of a clear tube. Although children aged 3–4 are able to reproduce this action after watching a demonstration (Cutting et al., 2011; Gönül et al., 2018), most children below the ages of 7–8 are unable to successfully innovate the hook. Even 10–11-year-olds do not exhibit mature levels of hook use (Beck et al., 2011; Cutting et al., 2011, 2014; Gönül et al., 2018; Whalley et al., 2017; but see Sheridan et al., 2016). Children similarly struggle to innovate in tasks requiring them to create a functional stick tool, meant to push a reward out of the middle of a horizontal clear tube, by unbending, combining or taking apart non-functional precursors (Cutting et al., 2011; Neldner et al., 2019). These findings have been replicated in several small-scale societies; recently settled San hunter–gatherer, Indigenous Australian and NiVanuatu agriculturalist children exhibit low rates of tool innovation in experimental paradigms (Neldner et al., 2017, 2019; Nielsen et al., 2014).

There are several methodological reasons why tool innovation tasks, like the hook task, might occlude the true breadth of children's innovative potential. Empirical measures of innovation are ill-structured, and rely upon a great deal of prior information in order to reach the prescribed solution (Chappell et al., 2013; Cutting et al., 2014). For example, to appropriately solve the hook task, children must understand the physical affordances of the pipe cleaner and basket handle, remember and flexibly apply prior knowledge of hook use to a novel context, and have the cognitive and motoric dexterity to perform all of the required actions within a 1–3 minute testing period. Under these constraints, failure does not necessarily indicate a lack of innovative ability (Reader et al., 2016), but may be attributable to any number of gaps in prior information or simply an inability to access it fast enough. These issues are compounded in experiments conducted in small-scale societies, where children are less likely than WEIRD children to have manipulated the precursor materials prior to the experiment, making object affordances especially opaque. For example, in a remote village in the Republic of the Congo, a Bondongo fisher–farmer child responded to the hook task by requesting he be allowed to retrieve his fishing hook, suggesting that a lack of familiarity with the affordances of the pipe cleaner may have prevented his success (Pope, unpublished data).

Further, in order to avoid confounding innovation with social learning, most experimental studies are designed so that children are tested individually to prevent the use of socially acquired information. However, applying socially learned information to new settings is probably central to tool innovation in everyday contexts (Muthukrishna & Henrich, 2016). For example, after seeing a pre-formed hook, dyads were more successful than individuals at solving the hook task (Gönül et al., 2019). In another extractive foraging task, complex tool innovation was observed in groups of 3–4-year-olds, but not individuals (McGuigan et al., 2017). In cultural evolution studies, in which tasks are solved over multiple generations, larger group size has been associated with greater improvement to existing technologies (Derex et al., 2013), increased complexity (Muthukrishna et al., 2014) and higher solution rates (Kempe & Mesoudi, 2014). Several modelling and ethnographic studies also demonstrate that group size and inter-group contact are positively correlated with material culture diversity (Caldwell et al., 2016; Collard et al., 2013; Henrich, 2004; Kline & Boyd, 2010; Shennan, 2001). These results suggest that innovation, even in the strictest sense, does not occur in an information vacuum. Instead, social and physical environments may influence innovative proclivity (Ivcevic, 2009).

## Hunter–gatherer social and subsistence contexts

Hunting and gathering societies are commonly defined by a subsistence economy that primarily relies on non-domesticated resources obtained via hunting, fishing and foraging (Kelly, 1995; Lee & Daly, 1999). Until approximately 12,000 years ago and before the emergence of agriculture, all human societies hunted and gathered for subsistence. Today, hunter–gatherers inhabit diverse environments, and have been shaped, among other things, by a long history of relationships with agropastoralists, colonisation and nation-states (Guenther, 2007). Reliance on non-foraged food is increasing, owing to growing engagements with neighbouring societies, new economic opportunities, state intervention and restrictions on the use of wild resources (Reyes-García & Pyhälä, 2016). While we acknowledge that defining hunter–gatherers by their subsistence economy reflects eighteenth-century European classifications rather than local ones (e.g. Barnard, 2004), in the absence of better terminology, we make use of the term ‘hunter–gatherer’ throughout the text.

Anthropologists have pointed to distinctive cultural and social traits that are shared by many hunter–gatherer societies varying in geography, ecology and histories, and which are usually not shared with their immediate agrarian or pastoralist neighbours (Endicott, 2011; Finlayson & Warren, 2010; Hewlett et al., 2011; Lee & Daly, 1999; Schweitzer et al., 2000). These cultural values include high levels of egalitarianism, which allow equal access to resources to all group members (Woodburn, 1982); a tendency towards mobility in which people and/or entire dwelling sites move frequently (Kelly, 1983; MacDonald & Hewlett, 1999); sharing which maintains the redistribution of material resources and social relationships (Lavi & Friesem, 2019); respect for personal autonomy, including the freedom and independence of individuals (Gardner, 1991; Woodburn, 1982); and lastly, small residential groups (Bird-David, 2017) with large lifelong and intergenerational networks (Bird et al., 2019; Dyble et al., 2015). Many hunter–gatherers also exhibit shared features of infancy, childhood and adolescence. These include close physical contact with mother, indulgence towards infants, frequent nursing, co-sleeping, weaning around three years of age, four-year birth spacing, separation and stranger rejection, dense social contexts, primary care by the mother, more father care than in other societies, transition into a multi-aged, mixed-gender playgroup in middle childhood, little child responsibility for subsistence and childcare, and few restrictions on childhood and adolescent sexuality (Konner, 2005, 2016). Because most research on child development is conducted in WEIRD societies (Nielsen et al., 2017), studying hunter–gatherer childhoods can provide an alternative and diverse perspective on the role of children as innovators, and the social contexts that encourage innovative propensities to flourish. In this paper, we consider aspects of the social and cultural environment, as documented among many contemporary hunter–gatherers, as especially conducive to the development of innovative children and adolescents.

Specifically, hunter–gatherers rely on non-domesticated resources that shift in availability and abundance seasonally, yearly and across generations (Kelly, 1983). And yet many hunter–gatherers routinely view their environment as abundant and giving (e.g. Bird-David, 1990). This may be due to social and subsistence strategies that mitigate risk, including widespread sharing of resources (Lavi & Friesem, 2019; Lewis et al., 2014; Peterson, 1993) and high levels of mobility (Kelly, 1983; MacDonald & Hewlett, 1999). Behavioural flexibility and increased rates of innovation may also help mitigate resource fluctuation. Indeed, several modelling studies suggest that rates of innovation increase in fluctuating environments (Acerbi & Parisi, 2006; Fogarty et al., 2015; Fogarty & Creanza, 2017). An ethnographic survey of 20 hunter–gatherer societies conducted by Collard and colleagues (2005) further showed that communities living in environments with a higher risk of resource failure had more diverse toolkits. Ethnographic studies also suggest that some hunter–gatherer societies exhibit high interpersonal variance in beliefs and skill (e.g. Gardner, 1991). Since diversity probably results in better group-level problem solving skills because individuals can draw upon a breadth of different experiences (Post et al., 2009; Smaldino, 2014), interpersonal variability may be adaptive to hunter–gatherers because it allows societies to continuously develop diverse toolkits that are better suited to novel environmental circumstances. In what follows, we suggest that aspects of socialisation documented in many contemporary hunter–gatherer societies may foster interpersonal variation and innovation in early life. Specifically, we argue that an emphasis on learning through autonomous exploration, adult and peer teaching, play and innovation seeking in adolescence probably encourages the development of children's tool innovation capabilities, especially in the domain of subsistence. In order to make our case, we link these socialisation practices to existing psychological research focusing on the development of problem-solving skills.

## Socialising innovative capabilities

### Autonomous exploration

As mentioned, respect for individual autonomy is a central social value in diverse hunter–gatherer societies (Endicott, 2011; Gardner, 1991). In order to respect individual autonomy, people avoid telling others what to do, and all community members have the freedom to choose their actions, whereabouts, and social associations. This emphasis on autonomy structures children's learning and development (Gardner, 2000; Lavi, in press; Morris, 1982). Indeed, autonomy is encouraged from infancy. Studies among the Aka, Batek, Paliyan and Inuit suggest that indulgence in the form of frequent touching, holding and on-demand breastfeeding allows parents to wait for children's initiative before they respond, and thus, supports the development of autonomy (Briggs, 1979; Endicott & Endicott, 2014; Gardner, 1966; Hewlett, 1992; Hewlett et al., 2000). For example, Bird-David (2008) argues that Nayaka parents believe that babies feed themselves, rather than being fed by parents.

In diverse hunter–gatherer societies, parents rarely interfere with young children's activities, even when they play with sharp knives or near fires (Crittenden, 2016a; Harris, 1980; Hewlett, 1992; Lancy, 2016a, b; Lew-Levy et al., 2019b; Naveh, 2014). In addition, parents occasionally make toy versions of adult tools for children, including bows, arrows, spears, digging sticks, fishing lines and baskets (Crittenden, 2016a; Dira & Hewlett, 2016; Hewlett et al., 2011; Imamura, 2016; Neuwelt-Truntzer, 1981; Nishiaki, 2013; Thompson, 2003; Wallace & Hoebel, 1952). Among the Aka, parents also show infants how to use these tools (Hewlett, 1992; Hewlett & Roulette, 2016). From early childhood onwards, children are afforded extensive autonomy to explore their surroundings. By the age of three, Nayaka children circulate among relatives and experiment with tools at will (Lavi, in press). Tsimane children's independent daily travel distance increases with age (Davis & Cashdan, 2019, 2020). Exploration in childhood probably gives children opportunities to learn the causal affordances of their cultural toolkits, observe how parents and other community members use these objects and determine when and where these tools are used (Bjorklund & Gardiner, 2012; Davis & Cashdan, 2020; Lancy, 2016a, 2017; Riede et al., 2018).

### Adult and peer teaching

From an evolutionary perspective, teaching can be defined as ‘behaviour that evolved to facilitate learning in others’ (Kline, 2015, p. 6). This definition leads to the broad inclusion of several social learning activities, such as opportunity scaffolding, chore assignment, instruction, correction and negative feedback (e.g. Boyette & Hewlett, 2017; Hewlett & Roulette, 2016; Kline, 2015). While lesson-style didactic teaching is commonly observed in WEIRD societies and frequently exported to non-WEIRD societies in school settings (Rogoff et al., 1996, 2003), such out-of-context child-focused activities probably play a limited role in knowledge acquisition in small-scale societies (Lancy, 2010, 2016b). Instead, more subtle forms of teaching, which tend to be embedded within meaningful community activities, are abundant. For example, among the Baka, children position themselves in participatory situations, such as by assisting in butchering, where they can overhear and elicit teaching from adults (Sonoda, 2016a, b). Similarly, Christian and Gardner (1977) note that parents make efforts to induce a Dene child to learn by listening and observing adults. In most cases, however, it is believed that the learner decides whether – and to what – she or he listens (Bombjaková, 2018; Christian & Gardner, 1977).

When it comes to tool manufacture, in many cases parents teach through opportunity scaffolding, during which a caregiver provides a child with an object, but does not provide cues on how this object should be used (Hewlett & Roulette, 2016; see also Kline, 2016). For example, among the Gidra, parents give children well-made child-sized bows as gifts (Nishiaki, 2013). Gidra children are expected to discover how to reproduce these bows without direct intervention from adults, and begin to skilfully produce bows at around 14 years of age. Similarly, Aka adults frequently made net fragments available to children, possibly so that children could reverse engineer their manufacture (Neuwelt-Truntzer, 1981). Nayaka adults refrain from interfering and instructing when children experiment with trap setting, preferring to let children learn from their own errors (Naveh, 2016).

Object exploration may facilitate children's innovative capabilities. Bonawitz et al. (2009, 2011), working with WEIRD pre-schoolers, examined the role of pedagogy in children's propensity for exploration. In their experiment, demonstrators presented children with a novel toy which could be manipulated to produce hidden functions, such as a squeaking sound, music or flashing light. In one condition, experimenters demonstrated a single function of the toy, while in another condition, children were left to explore the toy themselves. Children who were left to explore the toy autonomously discovered more of the toy's affordances than those in the pedagogical demonstration condition. By facilitating tool exploration through opportunity scaffolding, parents may encourage the discovery of novel object affordances.

While *in situ* learning is the norm in most societies surveyed, not all knowledge can be acquired through direct experience. In such cases, storytelling may be central to knowledge transmission among hunter–gatherers (Scalise Sugiyama, 2011; Weissner, 2014). Stories often address recurrent problems, such as inclement weather, missed hunting opportunities and the violation of social norms and practices. By listening to stories, children acquire a cumulative body of knowledge that they would be unable to develop independently, and can generalise this knowledge to solve unfamiliar problems (Scalise Sugiyama, 2017). Further, by listening to stories, learners gain new information that they can experiment with, improve upon and incorporate into their behavioural repertoire (Scalise Sugiyama, 2017), leading to innovative ways of performing tasks over time.

Horizontal teaching via demonstration, commands, feedback and instruction also plays a central role in knowledge acquisition from middle childhood onwards. Several developmental studies in WEIRD societies suggest that peer learning and teaching, or what Tomasello et al. (1993) call collaborative learning, increases children's ability to solve novel tasks (see also Azmitia, 1988; Perlmutter et al., 1989; Rendell et al., 2011). For example, children are more likely to employ logical reasoning when discussing a task with a peer than an adult (Kruger & Tomasello, 1986; Tomasello et al., 1993). Peer teaching may be beneficial because it forces children to take on another's perspective and assume complementary roles, ultimately facilitating the incorporation of new problem-solving stances into children's repertoire (Damon, 1984; Kruger & Tomasello, 1986; Phelps & Damon, 1989). In school settings, collaborative learning has been shown to be generative, in the sense that, by sharing information with peers, children can produce new knowledge unknown to either peer, including in the domains of mathematical concepts, moralistic reasoning and Piagetian conservation (Ames & Murray, 1982; Forman, 1989; Phelps & Damon, 1989; Kruger, 1992). Modelling studies suggest that horizontal transmission is especially important in fluctuating environments because it ‘creates the conditions for exploring the space of possible behaviours and for the emergence of the new behaviours appropriate to the changed environments’ (Acerbi & Parisi, 2006, para. 4.3).

A small but growing body of evidence suggests that collaborative horizontal learning is central to knowledge transmission in hunter–gatherer societies. For example, in a study of teaching subsistence skills using structured behavioural observations of Hadza and BaYaka 3–18-year-olds, child-to-child teaching represented approximately 75% of the observed teaching interactions, even as adults were in visual or auditory range of surveyed children 57–69% of the time (Lew-Levy et al., 2020; see also Boyette & Hewlett, 2017). Among the San (Imamura, 2016; Imamura & Akiyama, 2016; Shostak, 1976, 1981), Chabu (Dira & Hewlett, 2016), Aka (Boyette & Hewlett, 2017; Hewlett et al., 2011), Baka (Gallois et al., 2015, 2017, 2018), BaYaka (Lewis, 2002; Salali et al., 2016, 2019), Kaytetye (Thompson, 2003), Jenu Kuruba (Demps et al., 2012), Batek (Lye, 1997), Agta (Hagen et al., 2016), Pitjantjatjara (Ilyatjari, 1991) and Hadza (Crittenden, 2016a), researchers report that hunting, tree climbing, navigation, fishing, tool manufacture, medicinal plant knowledge and foraging knowledge are learned from and with other children. Such collaborative learning may improve children's ability to find novel ways of producing material culture.

### Learning through play

Play makes up a large proportion of the time budgets of all juvenile mammals (Bekoff & Byers, 1992), and involves the combination and recombination of established patterns and actions, which, over time, contributes to behavioural flexibility (Bateson, 2014; Fagen, 1981). Since play usually emulates the behaviours of mature species, play arguably allows juveniles to practice adult behaviours in safe settings (Smith, 1982). Unlike other mammals, humans engage in a species-specific type of play, known as pretence play, which involves ‘the projecting of a supposed situation onto an actual one, in the spirit of fun’ (Lillard, 1993, p. 349). Cognitively, pretence play involves ‘a capacity to generate, and to reason with, novel suppositions or imaginary scenarios’ (Carruthers, 2002, p. 229). Thus, pretence play may lay the groundwork for innovative thinking and problem-solving skills (Carruthers, 2002).

Many scholars report that much learning occurs in the mixed-sex, multi-age playgroup in hunter–gatherer societies (see Konner, 2005, 2016 for review). Pretence play, and specifically, work-themed pretence play, makes up about 20% of hunter–gatherer children's play time (see Boyette, 2018 for review). During pretence, children emulate adult social behaviours and practise subsistence skills (Fouts et al., 2016; Gosso et al., 2007; Lew-Levy & Boyette, 2018; MacDonald, 2007; Morelli et al., 2003; Neuwelt-Truntzer, 1981). For example, children build small huts with hearths adjacent to adult camps (Bombjaková, 2018; Crittenden, 2016a; Flannery, 1953; Ilyatjari, 1991; Lewis, 2002; Lew-Levy et al., 2019a; Mackie et al., 2015; Neuwelt-Truntzer, 1981; Shostak, 1976; Thompson, 2003; Tonkinson, 1978; Vanstone, 1965). In these play camps, children emulate the sexual division of labour, with boys pretending to hunt or hunting small game such as rodents or birds, and girls pretending to, or actually, cooking small versions of meals in cooking pots or tin cans. Among the Hadza and BaYaka, cooked food will carefully be shared among all those present, following the conventions of adult sharing (Crittenden, 2016b; Crittenden & Zes, 2015; Lew-Levy et al., 2019a). Among the Mbuti, Turnbull (1978) suggests that children emulate recently observed adult fights during play, while coming to a different resolution than adults. While playing hunter and hunted, Nayaka children vocalised animals’ fears, feelings and emotions (Naveh, 2014). Through these early play experiences, children both develop the subsistence skills necessary for full participation in the family economy and learn the rules of moral and social engagement (Bird & Bliege Bird, 2002, 2005; Bliege Bird & Bird, 2002; Bock & Johnson, 2004; Boyette, 2019; Crittenden et al., 2013; Crittenden, 2016a; Gallois et al., 2015; Hewlett & Cavalli-Sforza, 1986; Lye, 1997; Tucker & Young, 2005). For example, Gardner (1966) argues that Paliyan children are socially skilled and independent by the age of eight, and economically independent between the ages of 13 and 14. Likewise, Harris (1980) describes Yolngu children between the ages of 6 and 8 foraging, fishing, and swimming away from the supervision of adults.

While some of the cognitive benefits associated with pretence play may be deferred to adulthood, several developmental studies conducted in WEIRD societies suggest that children's play improves their problem-solving skills in the short term (Pellegrini & Gustafson, 2005; Sylva et al., 1976). For example, 3–5-year-olds who had the opportunity to incorporate sticks and clamps into their free play prior to a problem-solving task were able to combine these objects in novel ways to retrieve chalk from a box more quickly than children who were shown how to clamp the sticks together by a demonstrator (Sylva et al., 1976). The positive effect of play over demonstration on children's problem solving was heightened in problem-solving tasks that required more complex solutions (Smith & Dutton, 1979). Since children are smaller and weaker than adults, they face different adaptive challenges when participating in foraging; size and strength are a greater constraint for children than adults, while free time is not (Bliege Bird & Bird, 2002; Riede et al., 2018; Tucker & Young, 2005). As a result, children in hunter–gatherer societies sometimes use distinct, child-specific technologies when participating in food collecting. For example, Hadza children set sticky traps for collecting weaverbirds, an activity not conducted by adults (Crittenden, 2016a). Mikea children target smaller and shallower *ovy* tubers ignored by adult foragers (Tucker & Young, 2005). Baka children use tools unique to the playgroup, such as slingshots and small bows for hunting birds, squirrels and mice (Gallois et al., 2017). Baka children also develop unique names for edible plants, birds and mice that adults do not recognise. It is possible that the extensive participation in pretence play not only prepares children for adult work, but also generates child-specific food-producing innovations.

### Innovation seeking in adolescence

Features of adolescence observed among several hunter–gatherer societies make this developmental period especially tailored to learning innovations (Hewlett, in press, 2013, 2016). First, since most basic competencies are acquired by early adolescence, older adolescents may seek out more knowledgeable models from whom they can learn specialised skills or refine previously acquired skills (Henrich & Gil-White, 2001; Hewlett & Hewlett, 2012), such as in the domains of basketry (Puri, 2013), hunting (Dira & Hewlett, 2016) and hide work (Erikson, 1939; Ohmagari & Berkes, 1997), and for the manufacture of skis, sledges and canoes (Jordan, 2014). Second, adolescents have more free time than adults; while adolescents can, and often do, participate in many aspects of subsistence and childcare, they are not required to do so (Hewlett & Hewlett, 2012). Compared with farmers, hunter–gatherer adolescence is characterised by greater sexual freedom (Hewlett & Hewlett, 2012; Konner, 2005). Furthermore, with excess time and an increasing desire to find mates, adolescents, and particularly boys, often travel long distances (Hewlett & Hewlett, 2012; MacDonald & Hewlett, 1999), which provide opportunities to encounter, or even seek out, innovations from afar.

Three studies by Hewlett (in press, 2013, 2016) working with the Aka and Chabu suggest that, indeed, adolescents are highly receptive to acquiring novel technologies and culture forms. Both Aka and Chabu adolescents identified innovations as modifications and recombinations of previous technologies, such as new trap, house, basketry or pottery designs. In addition, Chabu adolescents identified innovations as being sought after. Aka adolescents identified innovators as calm and wise, while Chabu adolescents identified innovators as hard workers, kind and generous. Thus, for both the Chabu and Aka, prosociality was a central characteristic of innovators. Both Chabu and Aka adolescents were willing to travel long distances to learn from particularly good and innovative teachers, usually via oblique transmission. In both societies, adolescents autonomously selected individuals from whom to learn innovations. Aka adolescents were keener to learn innovative behaviours than adults, and stated that one of the reasons they did so was to impress potential mates, and to lead a good life. Chabu adolescents more frequently listed self-sufficiency and attracting mates as reasons to learn innovations. Both Aka and Chabu adolescents stated that learning innovations, particularly in the realm of subsistence and/or trade, is beneficial for supporting their parents and/or a future family. These studies suggest that adolescence is an opportune and adapted period of development for learning technological innovations.

## Archaeological implications

In archaeology, a growing number of researchers are considering the role of children and adolescents in the production and reproduction of technological and cultural products (e.g. Lillehammer, 2010a, 2010b; Nowell, 2015, 2016; Tehrani & Riede, 2008), including innovations (Riede et al., 2018). However, identifying child and adolescent innovators in the past has been hindered by the resolution the archaeological record has to offer. As opposed to the contemporary ethnographic context where fine-grained social processes can be directly observed, the nature of the archaeological record only allows us to infer social processes through the prism of material evidence, which is incomplete owing to archaeological formation and taphonomic processes (see Kelly et al., 2019 for discussion). To elucidate invisible social processes, ethnoarchaeologists have, at times, used selective ethnographic examples to support universal models of the human past, or to represent people of the past as ‘premodern’ or ‘primitive’ (see Athreya & Rogers Ackermann, 2018; French, 2019; Gosselain, 2016 for discussion). In the present paper, we reject a direct analogy between contemporary hunter–gatherer societies and the day-to-day reality of past hunter–gatherers. However, by calling upon evidence from a wide array of ethnographic, experimental and psychological studies, archaeologists can broaden their interpretive framework for understanding the cultural and behavioural diversity of past societies. Here, we hope to show that ethnographic data from contemporary hunter–gatherer societies can enrich how archaeologists think about, and interpret the archaeological contributions of, children and adolescents as innovators. In what follows, we examine whether the socialisation practices described above can be interpreted from the archaeological record. We focus on archaeological sites attributed to *Homo sapiens,* in order to avoid current debates around the limits of comparison of ethnographic data to other species of *Homo* (French, 2019), as well as debates around the authorship of transitional industries (e.g. Higham et al., 2014; Negrino & Riel-Salvatore, 2018). Nonetheless, many of the perspectives discussed could be relevant to other species of *Homo* (e.g. Nowell, 2016; Spikins et al., 2014).

### Autonomous exploration

Numerous artefacts have been interpreted as child-sized tools. For example, sites from North America and Europe have produced complete spears associated with the burial of adolescents (Trinkaus & Buzhilova, 2018), miniature spear throwers from Thule cultures and the Oregon Coast (Losey & Hull, 2019; Park, 1998), harpoon and dart heads from Thule and Dorset sites (Kenyon & Arnold, 1985; Park, 1998; Park & Mousseau, 2003), as well as bows, arrows and projectile tips of varying materials from Paleoindian and Palaeolithic European sites (Dawe, 1997; Frison, 1970; Kenyon & Arnold, 1985; Langley, 2018; Park, 1998; Rosendahl et al., 2006). Some of these weapons and weapon components may be functional (e.g. Dawe, 1997; Kenyon & Arnold, 1985; Losey & Hull, 2019; Rosendahl et al., 2006), while others are deemed too small to have been functionally useful, and thus have been interpreted as toys used in imitative play (e.g. Kenyon & Arnold, 1985). Some artefacts may have been manufactured by children; poorly made arrows from Rosebud Creek in Montana were arguably made for and by children (Dawe, 1997). Atypical use-wear patterns on poorly and irregularly shaped points at Trollesgave in Denmark have been interpreted as showing that they were used by children for woodworking and meat cutting (Donahue & Fischer, 2015), suggesting that children participated in work activities at this site. Whether scaled-down functional tools, or toys, these objects could have facilitated enskilment in the manufacture and use of complex tools.

Another line of evidence for childhood autonomy comes from Australian and European cave sites. Finger flutings and footprints may have been made by children exploring caves, including dangerous and difficult to access areas, either without adults (Bednarik, 1986; Roveland, 2000; Van Gelder, 2015b), or with older members of a group (e.g. Romano et al., 2019). The ability to explore tools and sites may have provided children with the opportunity to familiarise themselves with the affordances of their material culture and environment. For example, although not direct archaeological evidence, in an experimental study, a child innovated bipolar knapping. Following a period of attempting but failing to imitate the adult producing bladelets by holding the core on their lap, the child proceeded to place the flint on the pavement, essentially bipolar knapping on an anvil, thereby providing a solution to problems with motor control and hand–eye coordination (Sternke & Sørensen, 2009). Such creative solutions to child-sized problems serve to illustrate the positive consequences of allowing independent exploration, potentially leading to innovation.

### Adult and peer teaching

Lithics comprise an important evidence base for highlighting potential teaching in the archaeological record. The production sequence for making stone tools, known as the *chaîne opératoire* approach to analysis, has often been operationalised to identify instances of pedagogy in past societies (e.g. Audouze & Cattin 2011; Fischer, 1990a, b; Grimm, 2000; Karlin et al., 1993; Pigeot, 1990; Takakura, 2013; Cunnar 2015). Some scholars proposed that the spatial distributions of lithic raw material, tools and production waste may reflect different skill levels, indicating that novices were directly instructed by, or sought input from, experts (e.g. Fisher 1990a, b; Grimm 2000; Karlin et al. 1993). For example, at Palaeolithic and Paleoindian sites in Europe, Japan and North America, archaeologists have interpreted lithic cores, knapping scatters and/or completed preforms and tools left at the site as being produced specifically for the purpose of teaching (e.g. Audouze & Cattin, 2011; Bodu et al., 1990; Cattin, 2010; Cunnar, 2015; Fischer, 1990a, b; Karlin et al., 1993; Simonet, 2009, 2012; Takakura, 2013). Academic cores (Johansen & Stapert, 2008), involving the expert production of blade manufacture, with all by-products left at the site, have been found at Hattoridai 2 (Japan) and Pincevent (France) (e.g. Bodu et al., 1990; Karlin et al., 1993; Takakura, 2013). Poorly executed preforms and debitage scatters, positioned in an arc around more expertly produced debitage, have been interpreted as face-to-face learning experiences in the Great Basin (USA) (Cunnar, 2015) and at Trollesgave (Denmark) (Fischer, 1990a, b).

At some sites, spatial patterns suggest that novice knappers were more spatially distant from skilled knappers, and therefore may not have received direct instruction at the time that novice knapping occurred. For example, at Etiolles (France), Pigeot (1990) argues that master knappers worked close to the hearth, while less experienced knappers were kept on the outer edges of the knapping workshop. Based on an analysis of lithic assemblages at Hattoridai 2 (Japan), Takakura (2013, p. 160) argued that ‘the activity zones of the skilled knappers and novice knappers were differentiated based on well-defined spatial rules’. At Solvieux (France), Grimm (2000, p. 64) interprets lithic products from Location 1 as suggesting that novice flintknappers ‘may also occupy a space of benign community neglect […] where they configure their own learning relations with other apprentices’. Such spatially distinct practice areas may have served as a setting for peer teaching. At some of these sites, authors make the case for multiple learning processes, including expert–novice teaching and spatially distinct novice practice areas (e.g. Grimm, 2000; Takakura, 2013).

It is important to note that the interpretation of social interactions based on spatial patterning should be treated very carefully, especially in Stone Age, Palaeolithic or Paleoindian sites. For example, Hammond and Hammond (1981) suggest that children might disrupt original spatial patterning when experimenting with knapping and while collecting raw materials and tools left on the activity's surface. The clearing away of debitage into ‘dump’ areas can also disrupt or eliminate evidence of original activity areas (e.g. Bodu et al., 1990). Furthermore, the archaeological resolution in such sites rarely enables us to unequivocally determine the contemporaneity of such deposits (Bailey, 2007). For instance, whether more skilful knapping happened alongside novice production or a few hours/days/months later can be difficult to determine given the archaeological resolution. It is also debatable whether poorly manufactured tools should be used as a direct evidence for inexperience (see discussion in Hovers et al., 2011).

Nonetheless, experimental archaeological studies can help researchers consider the social interactions involved during learning in the deep past (see d'Errico & Banks, 2015 for a conceptual framework on teaching in Palaeolithic archaeology). For instance, Tostevin (2019) argues that learning flint knapping requires close intimacy and sharing of time. An experimental study performed by Putt et al. (2014) suggests that novice knappers produced more efficient flakes in non-verbal lessons compared with novice knappers who received verbal instruction. Thus, while archaeological evidence for face-to-face learning and the involvement of didactic lesson-style teaching as opposed to non-verbal observations and trial and error remains elusive, the convergence of ethnographic evidence for participatory teaching reviewed previously and the experimental findings outlined here brings to the forefront the possibility that didactic lessons may not be necessary to the successful teaching of complex skills, and also compel us to consider the role of peer interactions during the acquisition of knapping skill.

Beyond lithics, European Upper Palaeolithic parietal art, particularly illustrations of animals depicting their behaviours, social structures, physiology, kill zones through placement of weaponry and movement may have served the purpose of educating younger members of community about animal ethology and hunting (Azéma & Rivère, 2012; Guthrie, 2005; Mithen, 1988). Cooney (2018) further argues that children may also have participated in creating parietal art from early ages. Lombard (2015) argues that making and observing parietal art may have been used as a tool to help children learn, and generalise from, the cumulative body of knowledge that makes up complex skills, such as hunting.

### Learning through play

Numerous artefacts have been interpreted as toys, including dolls, balls, a toy snow knife, miniature sleds, animal and human figurines, and a shell (Jacobi, 2004; Kenyon & Arnold, 1985; Langley, 2018; Park, 2006; Politis, 1998; Riede et al., 2018). Lithic assemblages can also be interpreted as evidence for flint knapping by ‘beginners’ as part of ‘play’, particularly in assemblages where evidence of unskilled flintknapping appears to be unstructured and/or highly variable (e.g. Bodu et al., 1990). Finger flutings, prints or stencils of hands, fingers and other body parts found in caves may represent children playing with clay, and possibly making of figurative art (Bednarik, 1986, 2008; Cooney, 2018; Cooney Williams & Janik, 2018; Groenen, 1988; Hallam, 1971; Romano et al., 2019; Sharpe & Van Gelder, 2006; Van Gelder, 2015a). Stone rings found in northeast Greenland may represent elaborate playhouses made by Thule children (Hardenberg, 2010). Using stone and other materials, children are believed to have constructed a range of miniature summer and winter houses, as well as doll houses, in which objects representing key items such as ‘meat’ or ‘blubber’ were included. These houses may be compelling examples of the aforementioned pretence play, which underpin a capacity for innovation.

Toys may have also been incorporated into children's pretence play. For example, thaumatropes are bone discs on which an image is engraved on either side. By attaching a cord thread to the centre, the disc can be flipped back and forth, creating an optical illusion of a moving picture, such as a running doe (Azéma & Rivère, 2012; Langley, 2018; Nowell, 2015; Riede et al., 2018). Riede et al. (2018) interpret these bone discs as potential optical toys used to entertain children while helping them learn animal gaits, and may have also helped novice fibre spinners learn to twist and turn thread, thus developing the manual skills necessary for producing cordage. Thus, thaumatropes may serve as an example of how play, learning, technology and innovation are intertwined. Whether autonomously or while participating in community events (Cooney Williams & Janik, 2018; Nowell, 2015), play was central to children's lived experience in the past, and probably represented an important avenue for learning.

### Innovation seeking in adolescence

Several archaeologists suggest that, owing to availability of raw material, skill acquisition may have been limited to specific seasons and/or locations (e.g. Eigeland, 2011; Milne, 2005; Simonet, 2009, 2012; Sternke, 2011). Such learning experiences may have been reserved for older adolescents. For example, on Baffin Island, chert is only available during the summer months. Milne (2005) argues that, during this time, older adolescents who were strong and skilled enough probably trekked inland carrying chert nodules some 10 km from the source to the workshop, and participated in goose hunting. Since raw material would have been abundant at the teaching workshop, adolescents may have had ample opportunity to observe, experiment with, and learn new knapping innovations from especially skilled teachers.

## Concluding Remarks

In this paper, we have reviewed the psychological and ethnographic evidence for hunter–gatherer children as tool innovators. We have argued that the developmental niche of many hunting and gathering societies is conducive to socialisation practices that encourage child and adolescent innovative capabilities. These socialisation practices include autonomous exploration, peer and adult teaching, play, and innovation seeking in adolescence. While few empirical studies have directly investigated children's innovations, we found compelling evidence for children as innovators of child-sized subsistence technologies (Crittenden, 2016a; Gallois et al., 2017), and adolescents as active acquirers of innovations (Hewlett, in press, 2013, 2016). In doing so, this paper contributes an ethnographically grounded perspective on the cultural contexts which may favour the emergence of innovation in childhood. Further, using archaeological examples, we have provided an interpretive framework through which to consider child and adolescent innovators, and have pointed to some of the methodological challenges associated with identifying complex social learning processes in the past.

This review points to several next steps for broadening our understanding of cross-cultural variation in childhood innovation generally, and among hunter–gatherers specifically. For psychology, while the hook task and variations thereof have helped researchers understand the cognitive underpinnings of innovation, the novelty of the materials and the strangeness of the testing settings make it difficult to conduct in small-scale societies. In order to empirically assess the ontogenesis of innovative capacities in children, our metrics themselves must become more innovative. In addition to reward extraction tasks, we might consider measuring innovation in more naturalistic settings. Studies focused on how children innovate both individually and in groups, with familiar and unfamiliar materials, and that are based on the adaptive relevance of the outcome, are needed.

Ethnographers working with hunter–gatherer children should pay close attention to aspects of child cultures. In particular, we do not know how games, tools and novel subsistence strategies are developed, and whether individual children or groups of children are the sources of these innovations. Further, it is unknown whether adults adopt innovations generated by children, or if these remain within the playgroup. Building on Hewlett's (in press, 2013, 2016) diligent work on hunter–gatherer adolescents as innovation acquirers, more work is also needed on the role of adolescents in the transmission and generation of innovations in hunter–gatherer societies. Ethnographers should also examine how the considerable variation within and between hunter–gatherer societies influences children's innovative capabilities. Children inhabiting different ecologies may face different risks, limiting their ability to explore their environments autonomously (e.g. Blurton Jones et al., 1994; Hawkes et al., 1995). Within societies, sex differences in behaviour may also influence children's autonomous exploration (Draper, 1975; Froehle et al., 2019; Neuwelt-Truntzer, 1981). Beyond hunter–gatherer societies, changing social and ecological environments, and individualism, may lead to high rates of childhood innovations in other subsistence strategies as well (e.g. Glowacki & Molleman, 2017; Greenfield et al., 2000). As state intervention and formal schooling become part of hunter–gatherer children's social worlds (e.g. Lavi, 2018; Pollom et al., 2020; Reyes-García & Pyhälä, 2016), understanding how subsistence strategy and cultural transitions affect children's social learning experiences and innovative capabilities is also paramount.

Finally, for archaeology, the study of site formation processes is essential to better understand the integrity of the archaeological context generally, and spatial patterning specifically. In addition, increasing the focus on the effects of skill level in experimental archaeology will further improve our ability to assess technologies and better understand the processes involved in learning to use them (e.g. Eren et al., 2016; Milks, 2019; Whittaker et al., 2017; Whittaker & Kamp, 2006). Experimentation using unskilled WEIRD adults, whose educational experiences may shape their ability and willingness to learn in more exploratory and autonomous ways, can only go part of the way towards better identifying children in the archaeological record. In addition to adults having been socialised to learn in particular ways, there are also multiple physiological differences including motor skills, strength and neurological functions (e.g. Ford et al., 2009; Voelcker-Rehage, 2008; Voelcker-Rehage & Willimczik, 2006). While ethical considerations can pose greater challenges in recruiting children for experimental studies, these challenges are not insurmountable. In particular, ethnoarchaeological research with hunter–gatherer children (e.g. Politis, 1998) can shed new light on how children play, learn and behave within their communities, and how these behaviours impact the formation of archaeological sites.

## Research transparency and reproducibility

No data are associated with this article.
